# The impact of phytohormones on the number and quality of flowers in *Crocus sativus*

**DOI:** 10.1186/s12870-025-06712-6

**Published:** 2025-05-23

**Authors:** Jing Chen, Shuhui Yang, Xiaodong Qian, Xingchang Zhang, Yuanyuan Tao, Jing Li, Xiaoyuan Xi, Liqin Li

**Affiliations:** 1 Huzhou Central Hospital, Fifth School of Clinical Medicine of Zhejiang Chinese Medical University, Huzhou, 313000 China; 2 Huzhou Central Hospital, Affiliated Central Hospital of Huzhou University, Huzhou, 313000 China; 3 TCM Key Laboratory Cultivation Base of Zhejiang Province for the Development and Clinical Transformation of Immunomodulatory Drugs, Huzhou, 313000 China

**Keywords:** Phytohormones, Saffron, Floral induction, Crocin biosynthesis

## Abstract

**Background:**

Phytohormones play pivotal roles in regulating floral development and secondary metabolite synthesis in saffron (*Crocus sativus* L.).

**Results:**

This study investigated the effects of gibberellin (GA), abscisic acid (ABA), cytokinin (CK), and strigolactone (SL) on floral differentiation, stigma quality, crocin yield, and endogenous hormonal dynamics. GA significantly accelerated floral bud differentiation and apical bud elongation during reproductive transition, increasing flower number by 23.5% compared to the control. While CK also enhanced flowering (17.6% increase), ABA and SL showed milder effects. Intriguingly, ABA treatment markedly elevated crocin content, boosting crocin 1 and 2 levels by 49.5% and 99.2%, respectively, and total crocin yield per corm by 1.7-fold-the highest among all treatments. Endogenous hormone levels were dynamically regulated, with GA and ABA treatments upregulating endogenous ABA. However, qRT–PCR analysis revealed downregulated expression of ABA biosynthesis genes (*ZEP* and *NCED*) under GA and ABA treatments.

**Conclusions:**

These findings highlight GA as the most effective hormone for increasing flower number and ABA as the optimal choice for enhancing crocin content. This study provides actionable insights for hormone-mediated agronomic strategies to simultaneously improve saffron’s ornamental and medicinal value.

**Supplementary Information:**

The online version contains supplementary material available at 10.1186/s12870-025-06712-6.

## Introduction

Saffron, derived from the dried stigmas of the perennial herb *Crocus sativus* L. (family Iridaceae), is renowned for its medicinal properties, which mainly include promoting blood circulation to remove blood stasis, cooling the blood to detoxify, and relieving depression and calming the mind [1]. Owing to its triploid chromosome composition, this plant propagates asexually through corm reproduction, yielding infertile progeny [2]. The flower of saffron is characterized by six tepals, three stamens, and three stigmas. The stigma of saffron (the central part of the flower and the female reproductive organ) is highly valued and extensively utilized as a medicinal herb, spice, coloring agent, and flavoring ingredient [3–5]. The yield and productivity of saffron crop is directly dependent on the number of flowers produced, making flower enhancement a crucial factor for improving overall crop output.

The flowering mechanism in saffron is a complex physiological process regulated by a sophisticated interplay of various endogenous and exogenous factors, with temperature being the most crucial environmental factor [6]. In recent years, research on the cultivation of saffron has primarily focused on the effects of temperature and humidity control on the flowering period of indoor corms and the quality of stigmas [7, 8]. However, despite the fact that phytohormones are also an endogenous way to regulate plant flowering [9–10], studies on regulating saffron flowering through the exogenous application of phytohormones remain relatively limited. Specifically, Singh et al. [11] reported that abscisic acid (ABA), indole-3-acetic acid (IAA), gibberellin (GA), and cytokinin (CK) influence floral induction and flower organogenesis by regulating flowering-related genes such as *LEAFY* (*LFY*) and *FLOWERING LOCUS T 3* (*FT3*). Similarly, Haghighi et al. [12] found that Methyl Jasmonate (MeJA) and 6-benzylaminopurine (BAP) treatments significantly impact saffron flowering regulation, potentially enhancing the flowering process by modulating the expression of flowering-related genes and altering key physiological indicators. It should be noted that previous studies have demonstrated that treatment with BAP and GA3 can significantly enhance flower yield [13]. However, given that saffron cultivation in China predominantly employs a two-stage cultivation method, the effects of phytohormone treatment during the indoor cultivation phase on the number of flowers in saffron remain uncertain.

Indeed, numerous studies have highlighted the crucial role of endogenous hormones in the flowering process of various plants. For instance, it is widely recognized that GA accelerates flowering in plants by regulating floral meristem identity genes in leaves and promoting the formation of floral meristems in the shoot apical meristem (SAM). The mechanisms underlying this regulation have been extensively studied and reviewed [9]. In contrast, ABA, as one of the most important antagonistic hormones to gibberellins, has an unclear role in flowering control. As previously reported, it has both positive and negative effects [14–16]. Meanwhile, CK generally promote flower formation in a wide range of plants, however, the specific types of CK involved in inflorescence bud differentiation can vary significantly depending on the plant species [17–18]. Additionally, research has shown that the perception of strigolactone (SL) by *DWARF14* enables the transcription factor *TOE1* to inhibit flowering [19]. The flowering process is intricately regulated by the complex interplay among various hormones, ultimately achieving a state of homeostasis that fine-tunes the flowering process in plants. Given the significance of saffron flowering, it is essential to investigate the impact of phytohormones on the flowering process, especially in terms of influencing the number of flowers.

The primary active compounds in saffron include crocetin esters and crocetin, which are responsible for its vibrant coloring properties. Picrocrocin imparts its characteristic bitter taste, while safranal contributes to its distinctive aroma [20–21]. Crocins and crocetin, which possess anti-inflammatory and antioxidant properties, are important bioactive components in saffron [22]. In our previous study (data unpublished), a combined transcriptomic and metabolomic analysis revealed a significant correlation between the synthesis of secondary metabolites such as crocin and plant hormone pathways, which has drawn our attention to plant hormones. ABA is an important plant hormone that is widely involved in plant growth, development, and stress responses. The biosynthesis of ABA primarily occurs via the zeaxanthin pathway, with two key genes being z*eaxanthin epoxidase* (*ZEP*) and *9-cis-epoxycarotenoid dioxygenase* (*NCED*) [23]. The biosynthesis of ABA is a finely regulated process, influenced by a variety of environmental factors. For instance, drought and high-salinity stress can induce the expression of the *NCED*, thereby increasing ABA synthesis [24–25]. In addition, ABA synthesis is also regulated by the levels of endogenous hormones within the plant [25]. Both ABA and crocin are derived from zeaxanthin. Consequently, the level of ABA synthesis may have a direct impact on crocin production. Another plant hormone, SL, is synthesized from carotenoids and secreted into the rhizosphere through key enzymes such as β-carotene isomerase (D27) and carotenoid cleavage dioxygenase (CCD7/CCD8) in the roots [26]. Given that both SL and crocin are derived from the same precursor, β-carotene, and are catalyzed by different isoforms of the same enzyme family (crocin by CCD2 and SL by CCD7/CCD8), it is highly likely that they are closely related. When selecting the optimal cultivation methods, it is crucial to take into account both the quantity of flowers produced by saffron and the quality of the stigmas, which is contingent upon the content of crocin. It is well known that phytohormones play a regulatory role in the synthesis of plant secondary metabolites [27]. For example, it has been demonstrated that 2-isopentenyladenine has significant effects on the yield of artemisinin in *Artemisia annua* hairy roots [28]. Furthermore, it has been demonstrated that treatment with BAP significantly increases the content of crocin, picrocrocin, and safranal in the stigma of saffron, with better effects than those of GA3 [13]. In summary, plant hormones not only play a significant role in the regulation of saffron flowering but also significantly affect the quality and yield of the stigma by influencing the synthesis of secondary metabolites such as crocin. Therefore, the rational use of plant hormones to regulate flowering and the synthesis of secondary metabolites is of great significance in optimizing saffron cultivation strategies.

This study aims to explore how phytohormone treatments applied to saffron corms during dormancy, under a two-stage indoor cultivation system, influence flower development and the biosynthesis of secondary metabolites through flowering. To this end, saffron corms were subjected to treatments with GA, ABA, CK, and SL at two distinct time points during dormancy. The optimal hormone treatment was identified through phenotypic comparisons. Furthermore, the mechanisms influencing the synthesis of crocin in saffron were preliminarily elucidated by analyzing endogenous hormone levels and the expression of relevant genes.

## Materials and methods

### Plant material

The saffron (*Crocus sativus* L.) corms used in this study were cultivated in Bozhou, Anhui Province (33.88°N, 115.79°E), a region characterized by a humid climate with moderate rainfall, distinct monsoon patterns, and abundant sunshine. The long-term climatic data indicate an average annual rainfall of 938 mm and a mean temperature of 16.8 °C. The saffron cultivation followed a two-stage planting method, with corms planted in October 2023 and harvested in May 2024. After harvesting, the corms were categorized by weight for the experiment. In this study, corms with a weight range of 18–22 g, which were considered capable of flowering, were used. A total of 100 corms were randomly selected for each experimental group, and the number of flowers was subsequently counted. The stigmas were collected during flowering for quality and gene expression analysis.

### Hormonal treatment

A hormonal solution of GA (5 mg/L), ABA (5 mg/L), SL (5 mg/L) and CK (5 mg/L) was prepared in distilled water. One milliliter of each solution was applied to the apical bud of the corms and allowed to be absorbed naturally. The control samples were treated with distilled water. The first treatment was conducted on June 17th, and the second treatment was carried out two weeks later. The corms were cultivated indoors at an environmental temperature of 25 °C to induce flowering. The apical bud tissues of the corms were collected for morphological observation after 3 days of the last treatment. And the length of the apical buds was determined after 50 days of the last treatment.

To investigate the effects of hormones on the number and quality of flowers, on the day of flowering, the number of flowers in each group was counted, and the length and weight of the stigma were recorded. The stigma from the first flower that opened on each corm was collected, frozen in liquid nitrogen, and stored at -80 °C for future use.

### Determination of coloring, aroma, and flavor strength

To determine the strength of coloring, aroma, and flavor in stigmas -primarily attributed to the contents of crocin, safranal, and picrocrocin, respectively-sample preparation was carried out according to the procedure ISO-3632 [29–31], but saffron and solvent amounts were reduced proportionally. Initially, approximately 50 mg of saffron stigma were finely ground in a mortar. Subsequently, 10 mg of the ground sample was placed in a 20 mL volumetric flask containing 18 mL of distilled water. This suspension was stirred magnetically for 1 h in the dark before being topped up to 20 mL. A portion of the resulting aqueous extract was diluted tenfold and filtered through a 0.45 μm cellulose filter (Merck Millipore Ltd, Bedford, Massachusetts, USA). The UV-vis spectra of the filtered extract were then measured at 440, 330 and 257 nm using a Bio-rad smartspec plus spectrophotometer (Bio-rad, Hercules, California, USA) with a 1 cm quartz cuvette, employing pure water for blank correction. The spectra were captured with a resolution of 1 nm. The intensity values are expressed according to the following formula: $\:{A}_{1\%}^{1\:cm}$ (λ_max_) = (D * 20,000) (100 − H), where D is the absorbance at 257, 330, and 440 nm; 20,000 is the dilution factor of the total extract considering the amount of saffron sample; H is the content of moisture and volatile substances, expressed as a mass fraction.

### Analysis of crocins by UPLC-MS/MS

The UPLC-MS/MS was carried out on a Jasper UPLC system (SCIEX) and an AB SCIEX Triple Quad™ 4500 mass spectrometer (SCIEX) with an electrospray ion (ESI) source. The UPLC separation was conducted on a Phenomenex chromatographic column (3.0 mm × 100 mm, 2.6 μm, PFP 100 Å) maintained at 40 °C. The mobile phases consisted of an aqueous ammonium acetate solution (0.5 mmol; mobile phase A) and methanol (mobile phase B), which were used to separate crocin 1 and crocin 2. The separation was achieved at a flow rate of 0.5 ml/min under gradient elution conditions. The gradient conditions were: 0–1.3 min, 20% methanol; 1.3–4 min, 20–98% methanol; 4–5.7 min, 98% methanol; 5.7–5.8 min, 98–20% methanol; 5.8–6.8 min, 20% methanol. The injection volume was set at 5 µL. In multi-reaction monitoring (MRM) mode, crocin 1 and crocin 2 were detected using positive-ion scanning.

### RNA extraction, cDNA preparation and qRT-PCR

In order to study the genes related to ABA synthesis, namely *ZEP* and *NCED*, we aligned our stigma transcriptome data with the reported saffron genome [32] and removed genes with low expression abundance (FPKM < 1). As a result, we identified four *ZEP* homologous genes and three *NCED* homologous genes. Total RNA was extracted from stigma tissues using Takara MiniBEST Plant RNA Extraction kit according to manufacturer’s instructions. The cDNA was prepared using a verso cDNA synthesis kit (Takara, Japan) as per the manufacturer’s instruction. Expression analysis was done by using an ABI 7500 Real-time PCR system (Applied BioSystems, United States) using SYBR Premix Ex Taq (Takara, Japan) according to the manufacturer’s protocol. The *Tublin* was utilized as reference genes [33]. The 2^−ΔΔCT^ technique was used to calculate relative expression levels. Table 1 listed the primers that were used in this study.

**Table 1:** Genes and primers used in the qRT-PCR experiments

Genes	Gene_IDs	Forward PCR Primer (5′-3′)	Reverse PCR Primer (5′-3′)
*Tublin*	Csativus38860	CGTGCGTTTGTTCACTGGTA	CCCACCTCTTCGTAATCCTTC
*ZEP*	Csativus14656	CTGCGTCGTCTGCATCTTCTG	GCTGCCACAGAGTTCACTCCAT
*ZEP*	Csativus33005	ATGGGGATTCACGTCATATTGG	TGCCATGTCTCCTGGTCTAAGC
*ZEP*	Csativus58533	TCGGTGGTAGCAAGATGGTTAG	ATCCATGCCCGTAGTACTGCAC
*ZEP*	Csativus24340	CACCTGGACCACTTCCAAGC	GCATCGCCAGCTATGCATAC
*NCED*	Csativus31130	CCGGCAACGAAAGAACTATTC	CCGGCAACGAAAGAACTATTC
*NCED*	Csativus19183	GGTGGTGAAGGTGGACTTCGAG	CCCTCGTCATGCAAGTAACTCAC
*NCED*	Csativus43448	TCCCGCTTCGTCCAGACCTAC	CCCTCAGCAGGGTTCATCTGC

### Statistical analysis

Results were analyzed using one-way ANOVA, Kruskal-Wallis test or independent samples t-test by GraphPad software (significance level of *P* < 0.05; GraphPad URL: https://www.graphpad.com).

## Results

### The impact of different phytohormones on the differentiation and development of saffron

**Fig. 1:**
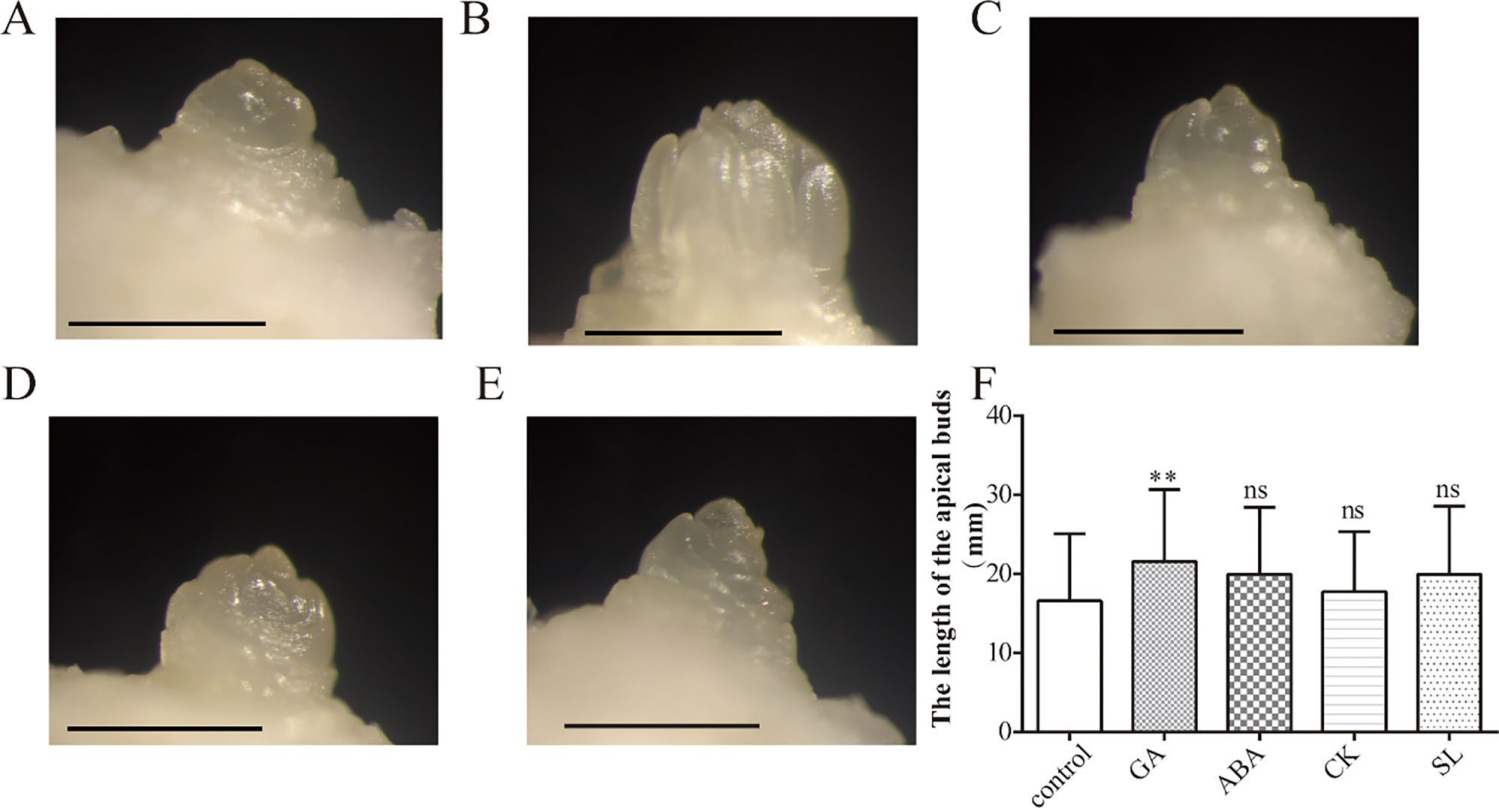
The effect of different phytohormone treatments on the growth state of saffron apical buds during the floral induction period and floral organ development period. **A**-**E**: Floral induction period (**A**: Control group; **B**: GA treatment group; **C**: ABA treatment group; **D**: CK treatment group; **E**: SL treatment group); **F**: Floral organ development period. Bar = 1 mm. Statistical analysis was performed using the Kruskal-Wallis test due to the non-normal distribution of the data. *P < 0.05, **P < 0.01, ***P < 0.001, **** *P* < 0.0001

The growth state of the apical buds of saffron during the floral induction period after treatment with different phytohormones is shown in Fig. 1A-E. Visual inspection of the images indicated that there are differences in the effects of different hormones on floral bud differentiation. Specifically, GA appears to promote the floral bud differentiation more effectively, accelerating its transition into the reproductive growth stage. In addition, ABA and SL also seem to have a slight promoting effect on floral bud differentiation, whereas CK does not show a clear promoting effect in these observations.

During the floral organ development period, the length of the apical buds in different hormone treatment groups was statistically analyzed. The results showed that the growth of the apical buds in each hormone treatment group was consistent with their effects on floral bud differentiation. Specifically, the apical buds in the GA treatment group exhibited a significant increase in length (*p* < 0.05), which suggests that GA markedly stimulates the growth of the apical buds, thereby hastening the development of floral organs. Although ABA and SL exhibited a slightly more pronounced effect on promoting apical bud growth compared to CK, the length of the apical buds in the ABA, SL, and CK treatment groups did not significantly differ from those in the control group (*p* > 0.05) (Fig 1F).

### The Impact of different phytohormone treatments on the number of flowers and the length and weight of the stigmas in saffron

**Fig. 2:**
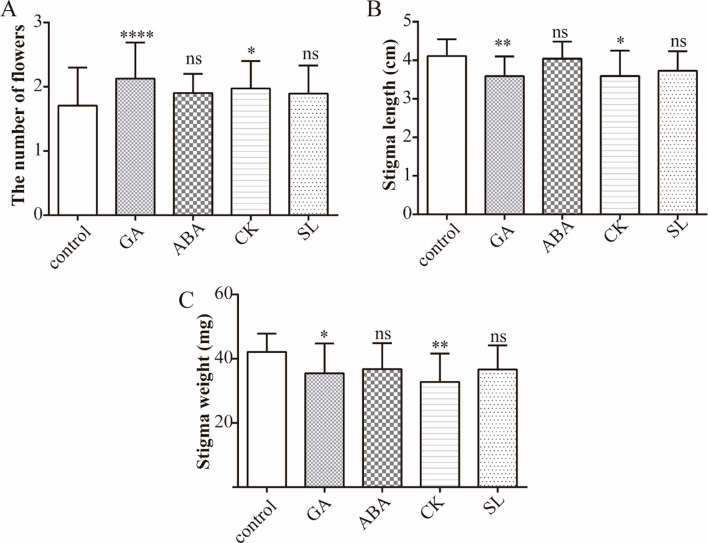
The effect of different phytohormone treatments on the number of flowers and the length and weight of the stigmas in Crocus sativus. **A**: Number of flowers; **B**: Stigma length; **C**: Stigma weight. Intergroup differences in flower numbers were analyzed by the Kruskal-Wallis test due to the non-normal distribution of the data. Intergroup variations in stigma length and weight were assessed by one-way ANOVA. *P < 0.05, **P < 0.01, ***P < 0.001, **** *P* < 0.0001

After treatment with different phytohormones, the number of flowers in saffron increased to varying degrees compared to the control group. Among them, the GA treatment group demonstrated the most significant increase in the number of flowers, with a rise from 1.7 to 2.1, which corresponds to an increase of approximately 23.5%. This indicates that GA has a remarkable efficacy in promoting flowering in saffron. Additionally, the CK treatment group also showed an increase in the number of flowers, increasing from 1.7 to 2.0, which corresponds to an increase of approximately 17.6%, thereby indicating that it has a certain promoting effect on flowering. In contrast, the ABA and SL treatment groups had a slight increase in the number of flowers, both reaching 1.9, which corresponds to an increase of approximately 11.8%, suggesting that these two hormones have a relatively weaker promoting effect on flowering in saffron (Fig. 2A).

Statistical analysis of the length and weight of the stigmas in each treatment group revealed that the length of the stigmas decreased compared to the control group after hormone treatment. However, there was no significant difference in the length of the stigmas between the ABA/SL treatment group and the control group. The weight of the stigmas was observed decreased in all treatment groups, with the CK treatment group showing the most significant reduction, followed by the GA treatment group. No significant differences were found between the ABA /SL treatment groups and the control group (Fig. 2B-C). Additionally, pairwise comparisons of each treatment group revealed that GA and CK had similar effects on flower number, stigma length, and stigma weight, while ABA and SL had similar effects (Table S1-S3). Overall, GA and CK significantly increased flower number but had negative impacts on stigma length and weight.

### The impact of different phytohormone treatments on the quality of the stigmas in saffron

**Fig. 3:**
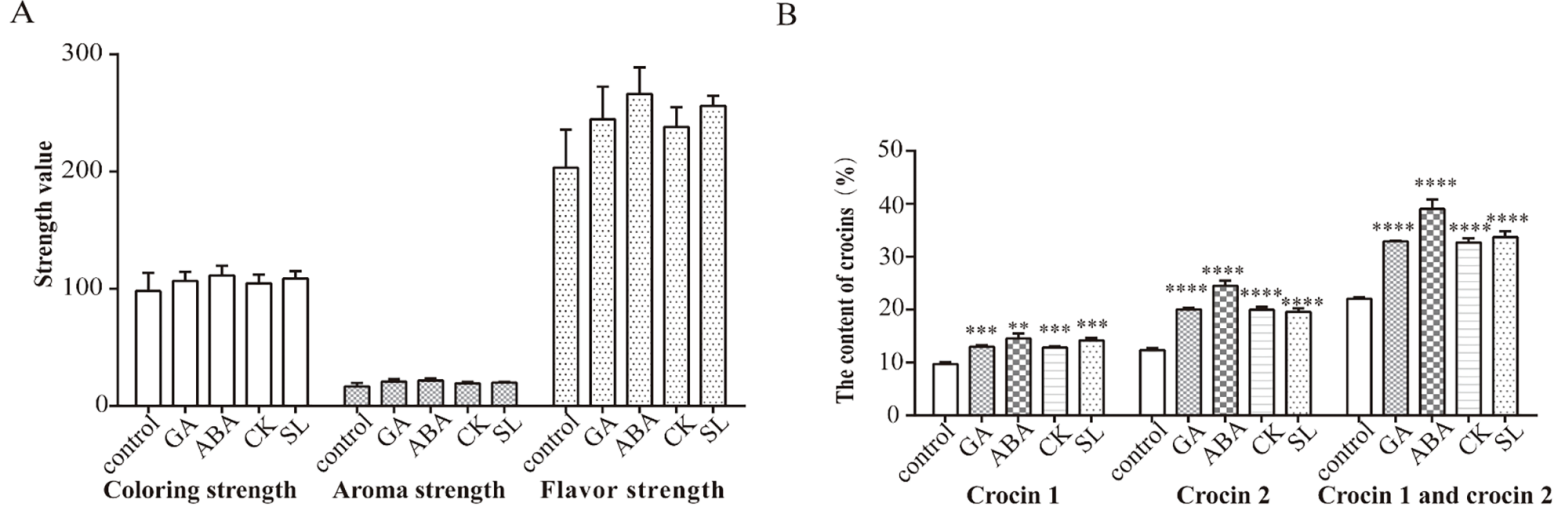
The effect of different phytohormone treatments on the quality of the stigmas in saffron. **A**: Strength of coloring, aroma, and flavor; **B**: Crocin content. Values (mean ± SD) were determined from three independent experiments (*n* = 3). Statistical significance was determined using an independent samples t-test. *P < 0.05, **P < 0.01, ***P < 0.001, **** *P* < 0.0001

After treatment with different phytohormones, the strength of coloring, aroma, and flavor of the stigmas were determined using ultraviolet spectrophotometry, and the content of crocin in the stigmas were assessed by UPLC-MS/MS. The results showed that strength of coloring, aroma, and flavor of the stigmas of each treatment group increased compared to the control group, although the differences were not significant. The accurate determination of crocin content revealed a significant increase in the content of crocin 1 and crocin 2 in the stigmas of all treatment groups. Notably, the ABA treatment group showed the most significant increase, with crocin 1 increasing from 9.7 to 14.5% and crocin 2 from 12.3 to 24.5%. The total content of crocin 1 and crocin 2 was enhanced to 1.8 times the original amount (Fig. 3).

### The impact of different phytohormone treatments on the yield of crocin in saffron

Crocin, one of the main components of saffron, possesses significant medicinal value, and the enhancement of its yield is of great importance for practical production. To analyze the increase in crocin yield in saffron after treatment with different phytohormones, we assessed the average amount of crocin produced per corm. The results showed that all treatments with GA, ABA, CK, and SL significantly increased crocin yield compared to the control group. Specifically, the increases in crocin yield were 1.7, 1.6, 1.5, and 1.4 times higher than the control group for ABA, GA, SL, and CK treatments, respectively (Table 2).

**Table 2:** The effect of different phytohormone treatments on the yield of Crocin

Group	Number of flowers per corm	The content of crocin(%)	Fresh weight of the stigmas (mg)	Moisture content of the stigmas(%)	Crocin yield per corm (mg)
Control	1.7	22.0	42.1	80.6	12.7
GA	2.1	32.9	35.5	80.6	19.7
ABA	1.9	39.0	36.8	80.4	21.9
CK	2.0	32.7	32.8	79.7	17.1
SL	1.9	33.7	36.7	79.2	18.6

### The impact of different phytohormone treatments on endogenous phytohormones and related genes

**Fig. 4:**
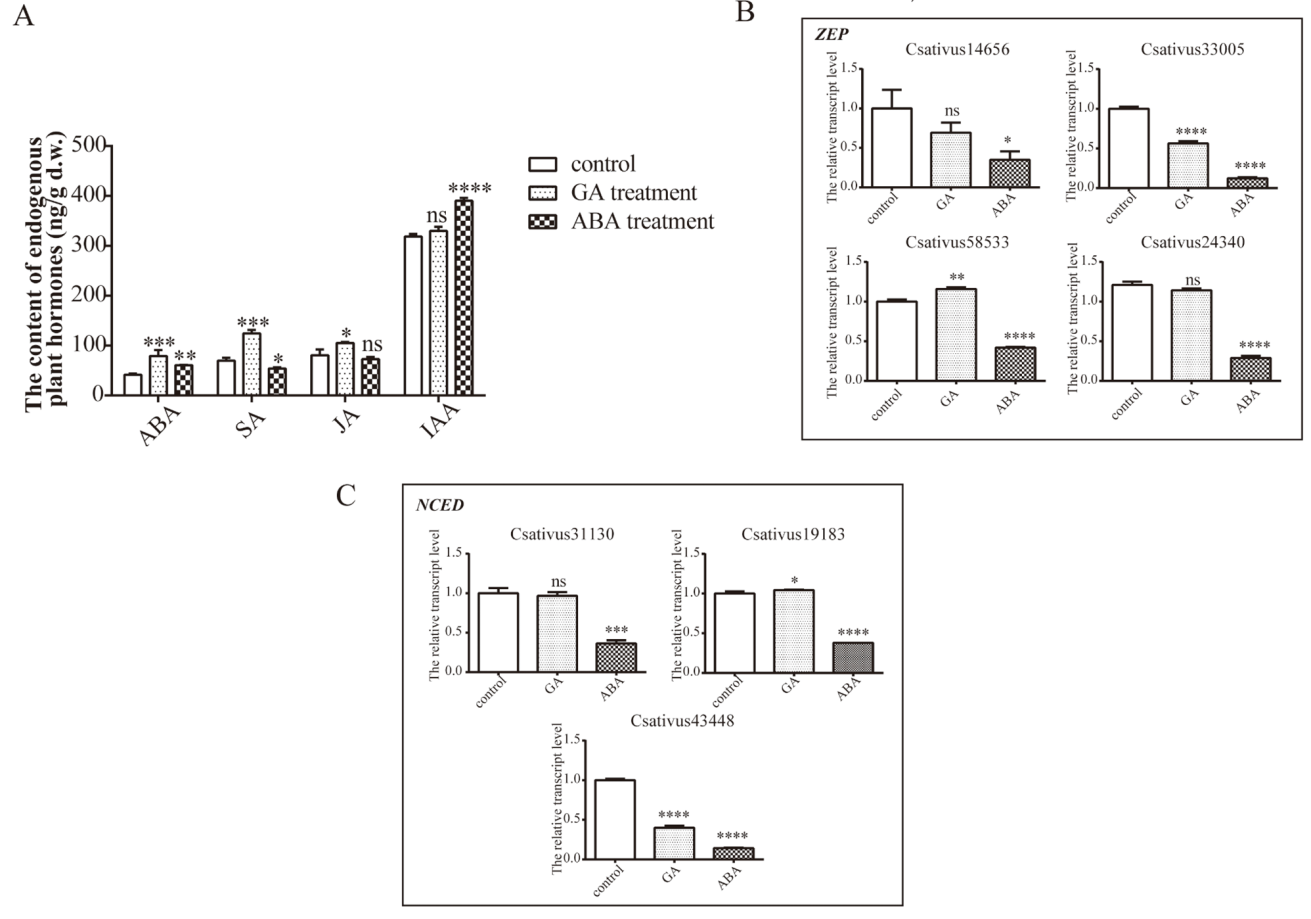
The effect of different phytohormone treatments on endogenous phytohormones and related genes. **A**: Endogenous phytohormones; **B-C**: ABA synthesis-related genes. Values (mean ± SD) were determined from three independent experiments (*n* = 3). Statistical significance was determined using an independent samples t-test. *0.01 ≤ *p* < 0.05; **0.001 ≤ *p* < 0.01; ***0.0001 ≤ *p* < 0.001; ****p < 0.0001

Endogenous phytohormones play a crucial regulatory role in the growth, development, and environmental adaptation of plants. To investigate whether hormonal treatments induce changes in the content of endogenous hormones, thereby affecting the number of flowers and the synthesis of crocin, we measured the levels of endogenous phytohormones ABA, salicylic acid (SA), jasmonic acid (JA), and IAA in the GA treatment group, which showed the most significant increase in the number of flowers, and the ABA treatment group, which exhibited the most notable increase in crocin content. The results indicated that both GA and ABA treatments significantly increased the content of endogenous ABA. ABA treatment also significantly increased the content of endogenous IAA, while GA treatment led to significant increases in the content of endogenous JA and SA (Fig. 4A).

Furthermore, qRT-PCR was employed to quantitatively analyze the expression levels of the crucial genes *ZEP* and *NCED* involved in ABA synthesis. The results indicated that, compared with the control group, the expression levels of both *ZEP* and *NCED* were significantly downregulated in the ABA treatment group (*p* < 0.05), while in the GA treatment group, only *ZEP* (Csativus33005) and *NCED* (Csativus43448) were significantly downregulated (*p* < 0.05), and the degree of downregulation was less pronounced than in the GA treatment group. This suggests that the pathway for ABA synthesis from zeaxanthin may have been inhibited in both ABA and GA treatment groups, with a more significant inhibitory effect observed in the ABA treatment group. Since ABA is merely a byproduct in the metabolic pathway from zeaxanthin to crocin synthesis, its reduced synthesis likely redirected more metabolic flux towards crocin production, thereby significantly increasing crocin content (Fig. 4B-C).

## Discussion

### Regulation of endogenous hormones and gene expression

**Fig. 5:**
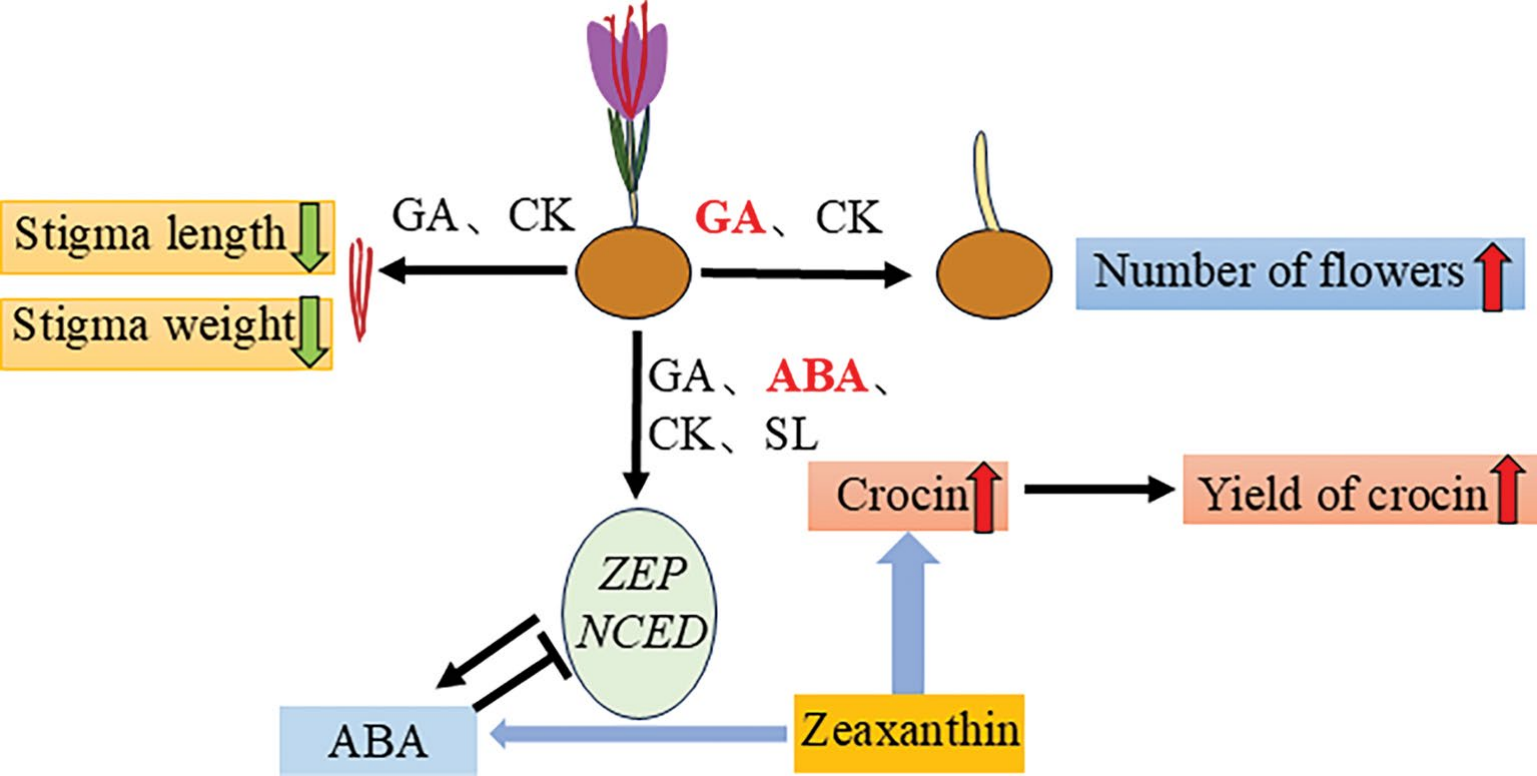
Mechanistic summary of the effects of hormones on flowering and crocin synthesis. GA and CK treatments increased flower number but reduced stigma length and weight, with GA having a more pronounced effect. All hormones (GA, ABA, CK, SL) increased crocin content, with ABA showing the most significant effect. Hormone treatments transiently upregulated *ZEP* and *NCED*, increasing ABA synthesis and endogenous ABA levels, which then inhibited *ZEP* and *NCED* via negative feedback, reducing ABA synthesis from zeaxanthin and redirecting metabolic flux towards crocin production. Green arrows indicate downregulation, red arrows indicate upregulation, and the thickness of the blue arrows represents the magnitude of metabolic flux. Note: The specific mechanisms depicted in this figure are based on current observations and hypotheses. Further experimental validation is needed to confirm these mechanisms

Although saffron, also known as “red gold”, is widely used, its production methods have remained largely unchanged since ancient times [34]. Developing cultivation techniques that enhance flowering quantity and improve the quality of stigmas is crucial for increasing saffron yield and its application value. The findings of the study have been summarized in Fig. 5, which demonstrate that various plant hormones exert significant effects on the differentiation and development of saffron, particularly in terms of floral bud differentiation, floral organ development, and crocin synthesis. Specifically, both GA and CK markedly enhance flower production, with GA exhibiting the most pronounced effect on flower quantity. All four plant hormones (GA, ABA, CK, SL) investigated significantly increase crocin content, with ABA achieving the most substantial elevation. The cause of this increase may be that after hormone treatment, the expression of key genes involved in ABA synthesis (namely *ZEP* and *NCED*) is transiently upregulated, leading to an increase in endogenous ABA levels. Then high levels of ABA may inhibit *ZEP* and *NCED*, and signal a decrease in ABA synthesis from zeaxanthin. Given that ABA is a byproduct in the pathway leading to crocin synthesis, this reduction likely steers the metabolic flux toward crocin production, thereby hiking crocin content.

In our study, we found that high levels of endogenous ABA may inhibit its biosynthetic genes *NCED* and *ZEP*. In fact, this negative feedback regulation is widely conserved across plant species. For example, in peanut (*Arachis hypogaea*), the AhNAC2-AhAREB1 protein complex mediates negative feedback regulation of ABA biosynthesis by inhibiting *AhNCED1* transcription, with AhAREB1 serving as the primary negative regulator [35]. Additionally, in Arabidopsis, the HD-ZIPII transcription factor AtHAT1 (homeodomain-leucine zipper protein 1) binds to the promoters of *AtABA3* and *AtNCED3*, suppressing both ABA biosynthesis and signaling [36].

ABA has two biosynthetic pathways: the terpenoid pathway and the carotenoid pathway. The latter is the main route for ABA synthesis. The key enzymes involved include *NCED* and *ZEP* [37]. Homologous genes of *ZEP* and *NCED* have been identified in saffron, but only the *CsNCED* gene has been extensively studied [38]. Our previous transcriptome data revealed that both *ZEP* and *NCED* are expressed in the stigma. The transcript levels of *ZEP* and *NCED* may be positively correlated with ABA synthesis. For instance, in potato tubers, ABA levels are regulated by the coordinated expression of *ZEP*, which supplies the substrate, and *NCED*, the rate-limiting enzyme [39]. Similarly, graphene oxide affects ABA levels by regulating ABA biosynthesis-related genes (*NCED*, *AAO*, and *ZEP*) in rapeseed (*Brassica napus* L.), thereby influencing root growth and development [40]. In this study, following GA and ABA treatments, the downregulation of *ZEP* and *NCED* genes in the stigma was noted, which may signal a decrease in ABA synthesis from zeaxanthin.

We speculate that the inhibition of ABA synthesis from zeaxanthin would lead to an enhancement of the metabolic flux towards the crocin pathway. Similar inferences have also been reported in other studies [32]. In our study, we found that all *NCED* and *ZEP* homologous genes were significantly downregulated in the ABA treatment group, indicating a substantial inhibition of the ABA synthesis pathway from zeaxanthin. Consistent with our previous hypothesis, this group exhibited the highest crocin content. Additionally, the metabolic trade-off between ABA and crocin production aligns well with our experimental findings - specialized drought treatments induced a 4.3-fold decrease in ABA content while simultaneously increasing crocin production by approximately 37% in stigma (data not yet published).

On the other hand, the results of this study show that both GA and ABA treatments increase endogenous ABA content, and ABA treatment also increases endogenous IAA levels. This indicates that exogenous hormone treatments can modulate the endogenous hormonal balance, which in turn affects plant growth, development, and the synthesis of secondary metabolites. Plant hormones exert their influence on the synthesis of secondary metabolites through a variety of mechanisms. For example, IAA treatment significantly increases polyphenol content by reactive oxygen species (ROS) in edible mushrooms [41]. Furthermore, ABA upregulates the expression of the ABA-responsive R2R3-MYB transcription factor *TcMYB29a*, which in turn activates the expression of taxol biosynthesis-related genes (such as *TcT5OH*), thereby promoting the accumulation of taxol in *Taxus chinensis* [42]. Therefore, the increase in endogenous hormones (IAA and ABA) caused by ABA treatment may also play a key role in promoting the synthesis of secondary metabolites, highlighting the complex interplay between hormonal regulation and secondary metabolite production in saffron. The specific mechanisms of action require further investigation.

### Impact of phytohormones on saffron flower development

The study revealed that GA has a pronounced promoting effect on floral bud differentiation and apical bud growth in saffron. This aligns with its well-documented role in accelerating flowering and reproductive development in various plant species, probably through the suppression of floral repressors such as DELLA proteins [43]. In saffron, the significant increase in apical bud length may be the result of GA promoting cell elongation, or it may be the result of GA promoting saffron to break dormancy and advance growth and development. This process could possibly be mediated by GA’s ability to modulate carbohydrate allocation and enhance cell division in the shoot apical meristem [13, 44–45]. However, further microstructural observations are needed to prove that GA promotes saffron floral bud differentiation and apical bud growth. Emerging evidence reveals GA’s dual role in floral development - while it initiates flowering through *LFY* activation, subsequent *LFY*-induced expression of *ELA1* (a cytochrome P450) inhibits GA4 biosynthesis by blocking C13-hydroxylation, thereby disrupting the GA-*DELLA*-*AP1* pathway essential for floral organ formation [46]. This paradoxical regulation may explain why saffron’s GA-treated buds accelerated floral initiation but subsequently showed compromised developmental progression, ultimately resulting in reduced stigma weight and length., suggesting the hormonal control of saffron flowering involves precise temporal coordination of both promotive and suppressive GA actions.

Although CK treatment showed only a minor direct effect on floral bud differentiation in saffron, the observed increase in flower number may be attributed to its indirect promotion of flowering through modulating hormone balance and enhancing vegetative growth. For example, CKs interact with the GA pathway, and this delicate balance between the two can be maintained by various proteins such as KNOX, SPY, and Sect. [47]. In addition, CKs can regulate sucrose metabolism, and the interplay between CKs and sucrose metabolism plays a significant role in controlling flowering [48–49]. However, the trade-off between increased flower number and reduced stigma quality under CK treatment indicates that while CK promotes floral initiation, it may simultaneously divert resources away from optimal stigma development. This highlights the complex balance between vegetative growth and reproductive investment in saffron, where CK appears to shift resource allocation toward flower production at the expense of individual flower quality.

In our study, we observed that ABA and SL had minimal impact on floral bud differentiation, bud development, and flower number in saffron, as well as no significant effect on stigma length and weight. This suggests that ABA and SL may play a relatively limited role in the flower development process of saffron, or their mechanisms of action differ from those of other hormones such as CK and GA. ABA is known for its role in stress responses and growth regulation, but the impact of ABA on flowering may hinge on a variety of factors. For instance, in rice, ABA can both promotes and suppress flowering, contingent upon the intensity of the stress and the genotype. SLs typically suppress axillary bud outgrowth [50], but their negligible effect on saffron’s flower number suggests its floral induction is decoupled from branching control. In summary, our findings highlight the complex interplay between different hormones in regulating saffron flower development, with GA and CK exerting significant effects, while ABA and SL appear to have limited influence, underscoring the intricate hormonal regulation and resource allocation strategies in saffron.

### Effect of phytohormones on stigma quality and crocin yield

Generally, the quality of saffron stigmas can be evaluated using UV spectrophotometry to measure color intensity, aroma, and flavor strength [29], along with UPLC-MS/MS to quantify crocin content. Notably, the coloring strength values obtained by UV spectrophotometry largely correlate with crocin content. Our study revealed that phytohormone treatments significantly improved saffron stigma quality. Importantly, while both UPLC-MS/MS-based crocin quantification and spectrophotometric analysis showed consistent trends, only UPLC-MS/MS detected statistically significant differences between treatment and control groups, attributable to two methodological advantages: (1) Selectivity and Specificity: The crocin content measured by UPLC-MS/MS corresponds to the coloring strength determined at 440 nm by spectrophotometry. However, UPLC-MS/MS employs MRM mode to detect specific fragment ions, thereby avoiding matrix interference at 440 nm that may affect spectrophotometric measurements. (2) Detection Sensitivity: UPLC-MS/MS demonstrates superior resolution in detecting low-concentration differences, enabling more precise quantification compared to spectrophotometry. In our research, the ABA treatment group showed the most significant increase in the content of crocin 1 and crocin 2, which are key bioactive compounds in saffron, holding significant medicinal and economic value. This may indicate that ABA plays a pivotal role in enhancing the synthesis of crocin through its own biosynthesis and degradation, signal transduction, and interactions with other hormones. Furthermore, the results indicate that all tested hormones (GA, ABA, CK, and SL) significantly increased the yield of crocin, with ABA showing the most pronounced effect. This is consistent with the observed increase in crocin content in the ABA treatment group. The enhancement of crocin yield by these hormones suggests that they could be used to improve the productivity of saffron cultivation. The differential effects of these hormones on crocin yield also highlight the importance of selecting the appropriate hormone or combination of hormones for optimizing saffron production.

## Conclusion

The findings of this study have important implications for saffron cultivation. The use of phytohormones, particularly GA and ABA, could be a viable strategy to enhance flowering and crocin yield in saffron. However, the observed reduction in stigma length and weight in hormone-treated groups warrants further investigation. Future studies should explore the optimal concentrations and application timings of these hormones to maximize their beneficial effects while minimizing any negative impacts on stigma development. Additionally, the molecular mechanisms underlying the hormonal regulation of crocin synthesis should be further elucidated to develop more targeted approaches for improving saffron quality and yield. In conclusion, this study highlights the significant role of phytohormones in regulating the growth, development, and quality of saffron. The findings provide a foundation for further research and practical applications in saffron cultivation, with the potential to enhance both the yield and quality of this valuable crop.

## Electronic supplementary material

Below is the link to the electronic supplementary material.

Supplementary Material 1

## Data Availability

Data is provided within the manuscript or supplementary information files.

## Abbreviations

GA: Gibberellin
ABA: Abscisic acid
CK: Cytokinin
SL: Strigolactone
IAA: Indole-3-acetic acid
LFY: LEAFY
FT3: Flowering Locus T 3
MeJA: Methyl Jasmonate
BAP: 6-benzylaminopurine
SAM: Shoot apical meristem
ZEP: Zeaxanthin epoxidase
NCED: 9-cis-epoxycarotenoid dioxygenase
CCD: Carotenoid cleavage dioxygenase
ESI: Electrospray ion
MRM: Multi-reaction monitoring
SA: Salicylic acid
JA: Jasmonic acid
ROS: Reactive oxygen species
