# Supplementary material for: The impact of phytohormones on the number and quality of flowers in Crocus sativus
Source: BMC Plant Biol. 2025 May 23;25:683. doi: 10.1186/s12870-025-06712-6 (PMC12100840; doi:10.1186/s12870-025-06712-6)
Supplement: Supplementary file 1 — Supplementary Material 1 [file 12870_2025_6712_MOESM1_ESM.docx]

**Table S1: Results of Dunn's Multiple Comparisons Test for Flower Number Following Hormone Treatment**

| Group Comparison | Dunn's Multiple Comparisons Test p Value | Significance Markers |
| --- | --- | --- |
| control vs. GA | < 0.0001 | **** |
| control vs. ABA | 0.1705 | ns |
| control vs. CK | 0.0119 | * |
| control vs. SL | 0.1901 | ns |
| GA vs. ABA | 0.0705 | ns |
| GA vs. CK | 0.6165 | ns |
| GA vs. SL | 0.0303 | * |
| ABA vs. CK | > 0.9999 | ns |
| ABA vs. SL | > 0.9999 | ns |
| CK vs. SL | > 0.9999 | ns |

**Table S2: Results of Turkey's Multiple Comparisons Test for Stigma length Following Hormone Treatment**

| Group Comparison | Turkey's Multiple Comparisons Test p Value | Significance Markers |
| --- | --- | --- |
| control vs. GA | 0.0080 | ** |
| control vs. ABA | 0.9938 | ns |
| control vs. CK | 0.0200 | * |
| control vs. SL | 0.1093 | ns |
| GA vs. ABA | 0.0233 | * |
| GA vs. CK | > 0.9999 | ns |
| GA vs. SL | 0.8634 | ns |
| ABA vs. CK | 0.0510 | ns |
| ABA vs. SL | 0.2370 | ns |
| CK vs. SL | 0.9122 | ns |

**Table S3: Results of Turkey's Multiple Comparisons Test for Stigma weight Following Hormone Treatment**

| Group Comparison | Turkey's Multiple Comparisons Test p Value | Significance Markers |
| --- | --- | --- |
| control vs. GA | 0.0494 | * |
| control vs. ABA | 0.2385 | ns |
| control vs. CK | 0.0043 | ** |
| control vs. SL | 0.1755 | ns |
| GA vs. ABA | 0.9792 | ns |
| GA vs. CK | 0.7824 | ns |
| GA vs. SL | 0.9828 | ns |
| ABA vs. CK | 0.5088 | ns |
| ABA vs. SL | > 0.9999 | ns |
| CK vs. SL | 0.4942 | ns |
